# Post intensive care syndrome - from risk at ICU admission to 3 months follow-up clinic

**DOI:** 10.1186/2197-425X-3-S1-A448

**Published:** 2015-10-01

**Authors:** J Torres, C Veiga, F Pinto, A Ferreira, F Sousa, R Jacinto, E Molinos, D Carvalho, C Dias, E Gomes, R Araújo

**Affiliations:** Unidade Local de Saúde de Matosinhos - Hospital Pedro Hispano, Matosinhos, Portugal; CINTESIS - Faculdade de Medicina da Universidade do Porto, Porto, Portugal

## Introduction

An increasing number of Intensive Care Unit (ICU) survivors develop psychological, cognitive and physical morbidities that may persist for years. The Post Intensive Care Syndrome (PICS) includes these multidimensional impairments and is described both for patients and families.

## Objectives

The aim of our study was to explore relationships between patient characteristics and PICS at the different stages of recovery.

## Methods

We invite patients that stayed in the ICU for more than 2 days to the follow-up clinic at 3 months. Before that, we evaluate them at ICU admission for risk assessment, at ICU discharge for PICS components and in hospital's first week and hospital's discharge to monitor the PICS evolution. Data from our Follow-Up Clinic and registry was prospectively collected from January 2013 to February 2015 and includes demographics, SAPS II, duration of sedation, duration of mechanical ventilation or ICU stay and also PICS risk and components. To evaluate the psychological components of PICS “Hospital Anxiety and Depression Scale” and “Post-Traumatic Stress Syndrome 14 Questions Inventory” were applied to patients during hospital stay and at 3 months. We compared patients with PICS at ICU discharge with no PICS population. A p value of less than 0.05 was considered statistically significant.

## Results

We analyzed 355 patients, 62% were male with a median age of 63 years, median SAPS II of 40, and median days of sedation and ventilation were respectively 2 and 3. Median days in the ICU and hospital were 5 and 18 respectively. 87.6% had risk to develop PICS and 52.4% actually developed PICS (35% had delirium; 33% weakness) at ICU discharge. PICS was present in 42% of patients at one week and 22% of patients at hospital discharge. At 3 months PICS was present in 30% of cases (depression and anxiety 14%; 8% PTSD risk; 7% mobility issues). The presence of PICS implied attitudes namely Psychiatry consultation in 50% of the cases, Physiotherapy in 22% of cases and Chronic pain in 9 patients.

When compared to the population without PICS, the patients who developed any of the PICS components at ICU discharge (mainly weakness and delirium) had significantly more risk, were more severely ill at ICU admission and stayed longer in ICU and hospital. The PICS group also died more in the first 3 months after discharge and survivors had more difficulties in getting back to work and had more PICS at 3 months (mainly anxiety and depression).

## Conclusions

More than half of our patients developed any component of PICS during and after ICU and that was related to illness severity and length of sedation, ventilation and ICU stay. At 3 months follow up the psychological PICS component predominates and was related to the PICS at ICU discharge, namely with the presence of delirium. The better understanding of delirium and its determinants will be essential to prevent long term PICS.

